# Heterogeneous melting near the Thwaites Glacier grounding line

**DOI:** 10.1038/s41586-022-05691-0

**Published:** 2023-02-15

**Authors:** B. E. Schmidt, P. Washam, P. E. D. Davis, K. W. Nicholls, D. M. Holland, J. D. Lawrence, K. L. Riverman, J. A. Smith, A. Spears, D. J. G. Dichek, A. D. Mullen, E. Clyne, B. Yeager, P. Anker, M. R. Meister, B. C. Hurwitz, E. S. Quartini, F. E. Bryson, A. Basinski-Ferris, C. Thomas, J. Wake, D. G. Vaughan, S. Anandakrishnan, E. Rignot, J. Paden, K. Makinson

**Affiliations:** 1 Department of Astronomy, Cornell University, Ithaca, NY USA; 2 Department of Earth and Atmospheric Sciences, Cornell University, Ithaca, NY USA; 3 British Antarctic Survey, Cambridge, UK; 4 Courant Institute of Mathematical Sciences, New York University, New York, NY USA; 5 Center for Global Sea Level Change, New York University Abu Dhabi, Abu Dhabi, United Arab Emirates; 6 School of Earth and Atmospheric Sciences, Georgia Institute of Technology, Atlanta, GA USA; 7 Department of Environmental Studies, University of Portland, Portland, OR USA; 8 Department of Geosciences, Pennsylvania State University, State College, PA USA; 9 Environmental Studies, Lewis & Clark College, Portland, OR USA; 10 Department of Earth System Science, University of California, Irvine, Irvine, CA USA; 11 Center for Remote Sensing and Integrated Systems, University of Kansas, Lawrence, KS USA

**Keywords:** Cryospheric science, Physical oceanography, Physical oceanography

## Abstract

Thwaites Glacier represents 15% of the ice discharge from the West Antarctic Ice Sheet and influences a wider catchment^1–3^. Because it is grounded below sea level^4,5^, Thwaites Glacier is thought to be susceptible to runaway retreat triggered at the grounding line (GL) at which the glacier reaches the ocean^6,7^. Recent ice-flow acceleration^2,8^ and retreat of the ice front^8–10^ and GL^11,12^ indicate that ice loss will continue. The relative impacts of mechanisms underlying recent retreat are however uncertain. Here we show sustained GL retreat from at least 2011 to 2020 and resolve mechanisms of ice-shelf melt at the submetre scale. Our conclusions are based on observations of the Thwaites Eastern Ice Shelf (TEIS) from an underwater vehicle, extending from the GL to 3 km oceanward and from the ice–ocean interface to the sea floor. These observations show a rough ice base above a sea floor sloping upward towards the GL and an ocean cavity in which the warmest water exceeds 2 °C above freezing. Data closest to the ice base show that enhanced melting occurs along sloped surfaces that initiate near the GL and evolve into steep-sided terraces. This pronounced melting along steep ice faces, including in crevasses, produces stratification that suppresses melt along flat interfaces. These data imply that slope-dependent melting sculpts the ice base and acts as an important response to ocean warming.

## Abstract (web summary)

Thwaites Eastern Ice Shelf observations from a new underwater vehicle show that high melt rates occur where ice is sharply sloped at the ocean interface, with lower melt where the ice is comparatively flat.

## Main

**Fig. 1:**
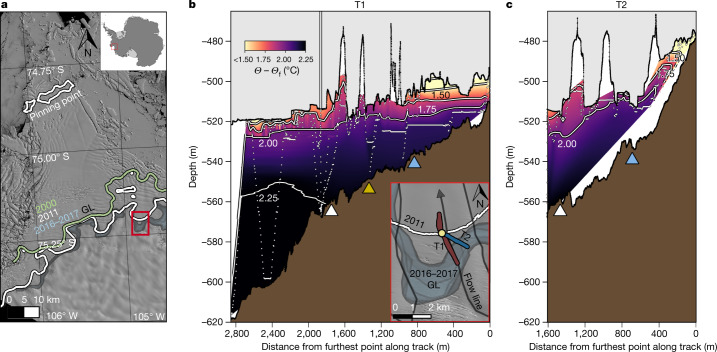
Warm water reaches near the ice base and retreating GL of the TEIS. **a**, Historical GL positions (coloured lines/zones after ref. ^12^) demonstrate notable GL retreat over the past two decades (QGIS map: Landsat 8, 15 m pixel^−1^, band 8 image LC08_L1GT_003113_20200131_20200211_01_T2_B8, 31 January 2020; the red box denotes the study region). **b**,**c**, Warm water is delivered close to the ice base (upper grey regions), shown by contours of thermal driving (degrees above in situ freezing point). The ice (black line) and seabed (brown regions) elevation profiles are measured by up and down altimetry from Icefin, which compare with bathymetry from mapping and forward sonar (Fig. 2). The small circles denote the Icefin track, along two transects approaching the GL, T1 (red) and T2 (blue) shown in the lower inset (red box from **a**). The yellow circle in the inset and vertical line through the ice denote the location of the borehole. The T1 track is oriented 5–10° oblique to the flow direction of the glacier and T2 approximately 50° oblique to flow; Icefin reached the grounded point of the glacier at the end of T2. Triangles in **b** and **c** mark historic GL locations estimated from satellite interferometry for 2011 (white), and the furthest downstream estimate in 2016 (blue)^12^. In **b**, the yellow triangle denotes the potential GL wedge detected by Icefin (Fig. 2). Nearest to the GL, although temperatures are colder than the deep water, the ocean water holds more than one degree of thermal driving. The ice base transitions from rough near the GL to terraced (progressively steeper-sided step-like features) near and downstream of the borehole, suggestive of progressive melting. Crevasses also contain terraces, especially clear in **c**.

Offshore ocean and atmospheric conditions force warm circumpolar deep water (CDW) onto the Amundsen Sea continental shelf^13,14^, where it contributes to ice loss and GL retreat of glaciers draining this sector of the West Antarctic Ice Sheet, including Thwaites Glacier^11^. Thwaites Glacier extends seaward from the Walgreen Coast, forming the Thwaites Glacier Tongue (TGT) to the west and the TEIS that rests on a prominent sea-floor pinning point (Fig. 1a). Warm CDW flows towards the glacier along the coastline and through sea-floor channels^15–17^, where it drives melting. The bed underneath the upstream grounded ice deepens to a maximum of 2,300 m below sea level^4,5^, making it susceptible to large-scale retreat from ocean-driven melting^7^. Collapse of Thwaites Glacier, which itself represents more than half a metre of global sea-level-rise potential, could also destabilize neighbouring glaciers that account for a further 3 m of future sea level rise^4^.

Changes in the Thwaites system have accelerated over the past 20 years (refs. ^8–10^), resulting in breakup of the TGT and propagation of rifts across the TEIS^10^. Recent GL retreat has varied from 0.6 to 1.2 km year^−1^ (ref. ^12^). Ocean melting, dynamic thinning and ice-flow rates influence this retreat^12^, but exactly how these factors operate is difficult to constrain with generally poor observations below the ice. Satellite observations, which measure the surface elevation of the glacier, suggest that the TEIS is thinning on average 25 metres per decade^10,12^, whereas airborne ice-penetrating radar that directly measures ice thickness estimates rates up to 45 metres per decade^18^.

Although ocean-driven melting directly influences the stability of ice around Antarctica^19,20^, little data resolve the interaction between the ice and ocean directly^21–30^. Models of ocean forcing are often limited by resolution or available parameterizations. Generally, models represent ice shelves simplistically as wedges of ice with flat or curved interfaces and an inferred sea-floor geometry as a function of distance from the presumed GL. Usually a zero-melt condition is imposed at the GL^31^, which is inconsistent with evidence of thinning and GL retreat. Although retrograde bed slopes facilitate positive feedback in grounded ice loss from ocean-forced melt^6,7^, glaciers resting on prograde slopes still face influence from warm water undercutting the ice. Temperature and salinity variations influence circulation and heat exchange between the ice and ocean. These variations occur at scales much smaller than those resolved by remote sensing or captured in ice-shelf-wide models of ice–ocean interactions. Furthermore, few direct measurements have been made near the ice base^24–30^, and none at the GL of a considerable glacier, that would help models at large and small scales better represent melting. Therefore, how melting occurs under ice shelves and particularly at the GL, influencing ice loss, remains largely unresolved.

**Fig. 2:**
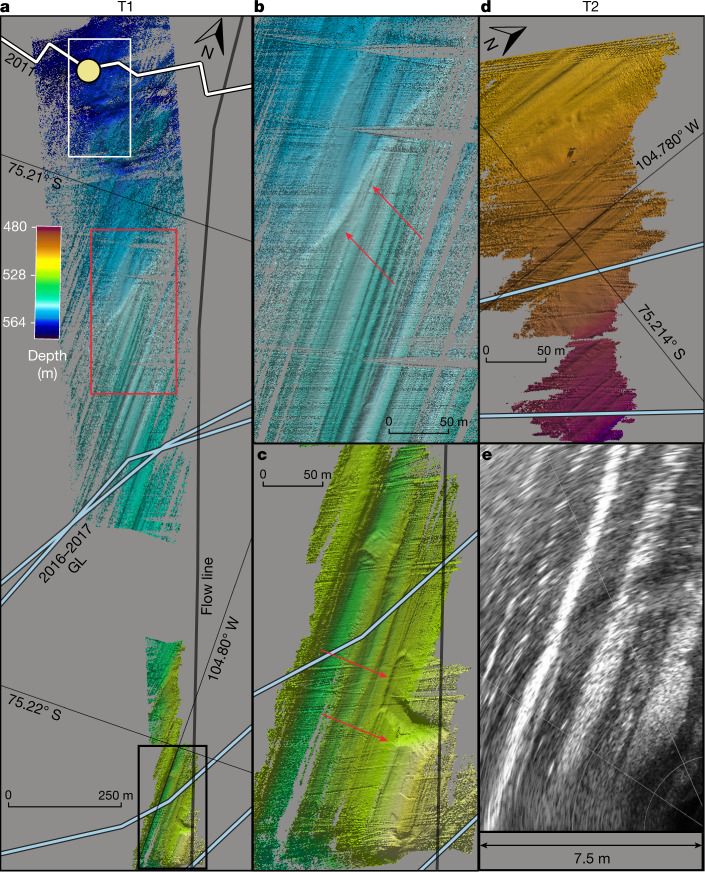
Sea-floor bathymetry shows smooth retreat and ice–bed interactions near the TEIS GL. The sea-floor bathymetry near the TEIS GL is characterized by along-flow ridged bedforms having several different wavelengths, as well as evidence for two possible former GL positions (white and red boxes) and channelized subglacial outflow (black box). Data in **a**–**d** are from downward-facing bathymetric sonar and **e** from forward sonar on Icefin. Reworked sediments (white box) are observed near the borehole (yellow circle). **b**, A single sinuous 2–3-m-tall slope consistent with a GL sediment wedge is found about 200 m north of the 2016 GL estimated from remote sensing (red boxed region from **a**; red arrows denote wedge). The wedge crosscuts the 2–5-m wavelength along-flow bedforms (Extended Data Figs. 5 and 6). **c**, An isolated 4-m-deep channel cut into the sea floor makes two sharp turns and includes a segment that cuts perpendicular to most bedforms, suggesting that this feature formed from rerouting of subglacial water as the GL retreated (Extended Data Fig. 5). **d**, The bedform topography near the GL of T2 shows evidence of linear ridges striking north (Extended Data Fig. 4). **e**, Forward-looking sonar data of the ice base near the GL shows that the ice has the same 2–5-m-wavelength ridges as the shortest-wavelength features on the sea floor. These data together suggest that GL retreat has been largely continuous over the observable period, since at least 2011 based on remote sensing. Moreover, the similarities between the bed and ice morphology at the GL suggest that the ice–bed interactions set up slopes that are then progressively melted by the intruding seawater. Bathymetric sonar, **a**–**d**, was processed in Qimera and projected using QGIS. Forward sonar are projected using the Oculus ViewPoint software.

**Fig. 3:**
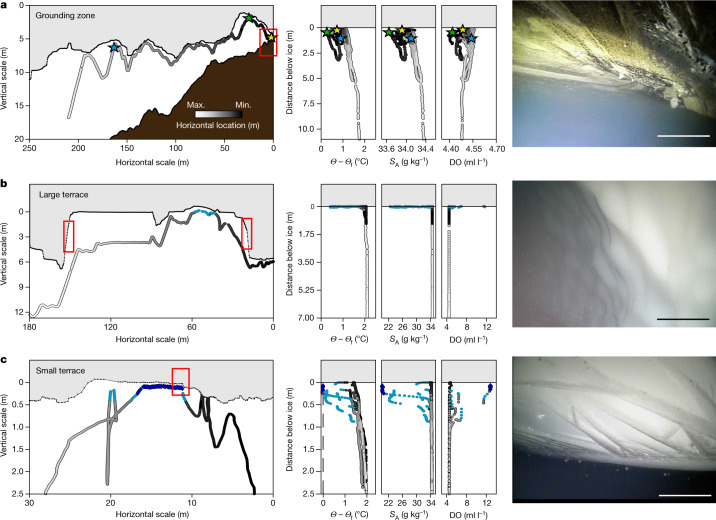
Ocean conditions influence ice base morphology, which varies with distance from the GL. The Icefin vehicle track is shaded by relative along-track distance from downstream (white) to upstream (black). Light-blue data denote regions with cooling and freshening in terraces and dark blue denotes the coldest/freshest data observed. **a**, Conditions in the near-GL water cavity show the influence of melting (freshening) close to the GL along T2 (left). Coloured stars denote close passes to the ice that also have distinct signatures of mixing and melting. Vertical profiles of thermal driving (*Θ* − *Θ*_f_), absolute salinity (*S*_A_) and dissolved oxygen (DO) binned with distance from the ice base show complex signatures that vary with location (Extended Data Fig. 3), suggesting the influence of both melting and SGW outflow (centre). Imagery near the GL (red box) shows ridged ice topography and sediment-laden clear basal ice at the GL (yellow star) (right). Scale bar, approximately 0.5 m. **b**, Ocean conditions in a large terrace formed in the ice base imply melting near the sidewalls (red boxes, 800 m from the GL along T2) (left). Warm, salty water (black, grey) is found along the sidewalls, whereas much fresher and more oxygenated water with low thermal driving (cold relative to in situ freezing) collects in the terrace roof (centre). Imagery of terrace sidewalls across the TEIS uniformly show scalloped surfaces reflecting turbulent melting (Extended Data Fig. 8 and Supplementary Video 1) (right). Scale bar, approximately 0.5 m. **c**, As in **b** but for a small terrace at 2,400 m downstream along T1 that contains cold, fresh and oxygen-rich water along its roof. Here the water becomes supercooled, with ice crystals forming laterally (right) across the heavily stratified interface (red box) between this 0.1 m upper boundary layer and the warm, saline and more oxygen-poor lower ocean waters. Scale bar, approximately 0.1 m.

**Extended Data Fig. 1:**
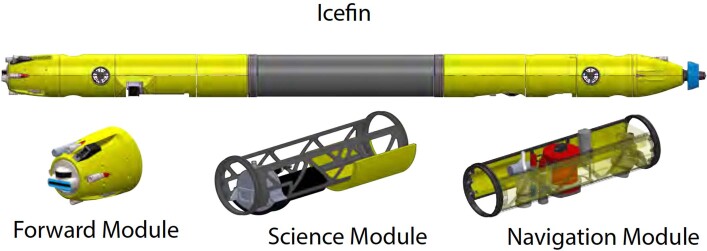
The Icefin vehicle is a modular underwater vehicle designed to deploy through boreholes in the ice (diagram produced using SolidWorks). The vehicle consists of a total of seven modules: forward science including oceanographic sensors (CTD, DO), forward-looking sonar, cameras and lighting; forward directional thrusters; customizable science payload, shown here as configured for the Thwaites missions with a bathymetric sonar; electronics module; aft science and navigation module with DVL/ADCP, altimeter, high-definition camera and light; aft directional thruster; and rear thruster with rear camera. The fibre-optic tether mounts to a bridle at the tail of the vehicle and connects through the rear bulkhead of the electronics module, delivering live data feeds to the surface.

**Extended Data Fig. 2:**
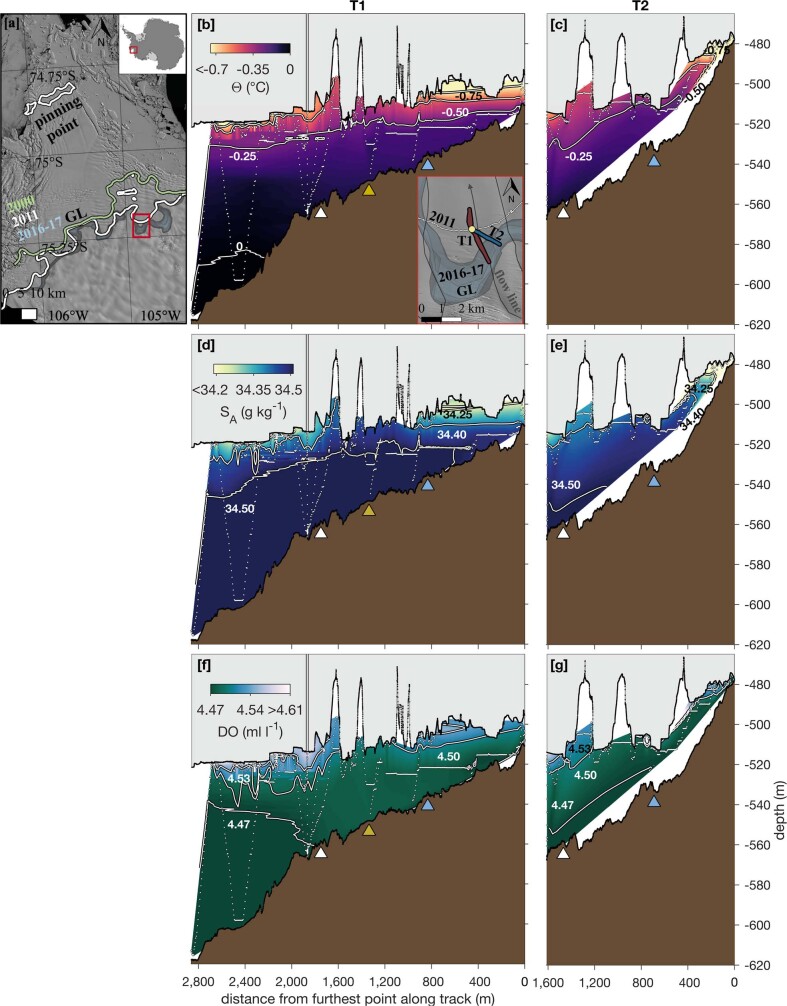
Salinity and dissolved oxygen generally track with temperature near the retreating GL of the TEIS. **a**, As in Fig. 1, image of the TEIS, with historical GL positions in coloured lines showing notable retreat over the past two decades (QGIS map: Landsat 8, 15 m pixel^−1^, band 8 image LC08_L1GT_003113_20200131_20200211_01_T2_B8, 31 January 2020; the red box denotes the study region); inset denotes the geographic location of the TEIS in relation to Antarctica. **b**–**g**, Hydrography of the ocean under the ice shows that absolute salinity (**d**,**e**) and dissolved oxygen (**f**,**g**) track with temperature (Fig. 1b,c) under the TEIS. The inset in **b** provides a focused view of the study region: the yellow circle denotes the location of the hot-water-drilled access hole, the red line represents T1 (5–10° oblique to the flow direction of the glacier) and the blue line represents T2 (50° oblique to flow). Triangles in **b**–**g** mark historic GL locations estimated from satellite data^12^ (white, 2011; blue, 2016–2017) and shown by the Icefin bathymetric sonar data (yellow).

As part of the National Science Foundation (NSF)-Natural Environment Research Council (NERC) International Thwaites Glacier Collaboration (ITGC), a comprehensive field campaign was carried out over two austral summers, with an ice-shelf-drilling campaign in 2019–2020 to access the ocean cavity^28^ and sediments below the TEIS to observe the changing system directly. We conducted detailed in situ hydrographic measurements in an area of the TEIS referred to as the ‘butterfly’^12^. The ice in this region is grounded at about 500 m below sea level (Figs. 1 and 2), typical of most of the Thwaites system outside the western trunk. We deployed the new underwater vehicle Icefin (Extended Data Fig. 1) through the borehole over five under-ice missions spanning 9–11 January 2020. The vehicle measured ocean temperature, salinity, dissolved oxygen and current velocities (Fig. 1 and Extended Data Fig. 2), mapped the sea floor and ice base in three dimensions (Figs. 1 and 2) and imaged the ice and sea floor (Fig. 3).

## Conditions under the ice shelf

**Extended Data Fig. 3:**
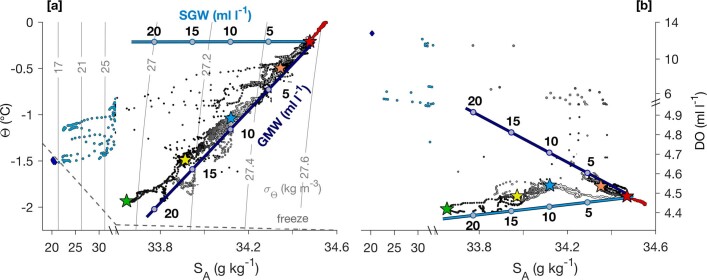
Salinity, temperature and dissolved oxygen profiles show signatures of melting and mixing below the TEIS. *T*–*S* (**a**) and DO–*S* (**b**) diagrams compare hydrographic data from T1 and T2. Data are colourized after the data shown in Figs. 3 and 4 (with distance along track and for which blue colours denote extremely fresh section) and stars denote locations also called out with stars in Figs. 3 and 4. The warm, salty and oxygen-poor data in red are not shown in Figs. 3 or 4 but come from the outermost data from Extended Data Fig. 2 at distances greater than 10 m from the ice base. This water does not interact with the ice base in the surveyed region. Heavy lines show linear mixing lines between the source water mass responsible for melting the ice base locally (red star) and a pure mixture of GMW or SGW under fully turbulent mixing conditions^35,37,42^. These may not fully describe sheltered environments along terrace roofs, in which diffusive processes may dominate^43,44^.

The ice base deepened with distance from the GL, ranging from about 500 m to 520 m below sea level over the nearly 3 km T1 survey (Fig. 1b) and sloping downward more steeply along T2 from a minimum of 475 m depth at the GL (Fig. 1c). The sea floor (prograde) also sloped downward away from the GL (Figs. 1 and 2). The temperature, salinity and dissolved oxygen content of ocean water reflects mixing of different reservoirs, including ocean, melted glacial ice (glacial meltwater (GMW)) and subglacial water (SGW) from beneath the grounded ice. Warm water occupied much of the ocean under the TEIS, with maximum thermal driving of 2.25 °C (ocean temperatures 2.25 °C above the in situ freezing point), decreasing only slightly to 2 °C within about 5–10 m of the ice base and 400 m of the GL (Fig. 1b,c). This decrease in thermal driving resulted from pressure release, a cooling of the water from 0 °C to −0.25 °C and a freshening from 34.50 g kg^−1^ to 34.40 g kg^−1^ (Extended Data Fig. 2). Dissolved oxygen, which reflects both exchange with the atmosphere before submerging below the ice and that released from melting ice, increased in concentration over this region from 4.47 ml l^−1^ to 4.50 ml l^−1^; the coupled change in hydrographic conditions indicates a slight increase in GMW closer to the ice (Extended Data Fig. 3). The relatively well-mixed water column was overlain by a stratified upper layer, generally 5–10 m thick, at which the ocean cooled, freshened and increased in oxygen owing to local ice melt producing a greater admixture of GMW.

**Extended Data Fig. 4:**
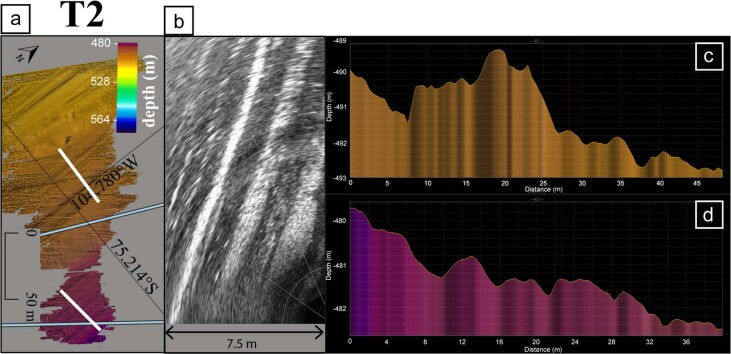
The bathymetry near the GL of the TEIS along the T2 survey is characterized by ridged bedforms of varying wavelengths. **a**, Sonar-derived bathymetry from nearest the GL, coloured by depth, showing examples of linear ridges (after Fig. 2, bathymetry and profiles produced with Qimera and projected in QGIS). White lines denote the position of profiles found in **c** and **d**. **b**, Forward-looking sonar shows that the ice-shelf basal topography near the GL (after Fig. 2) is characterized by similar ridges, having an approximately 2.5 m ridge-crest spacing and sloped faces. Forward-looking sonar is projected in Oculus ViewPoint. **c**,**d**, Linear profiles of the bed topography across the region show evidence for ridges with approximately 1-m, 2–2.5-m and 5-m wavelengths. These data show that the shape of the ice surface at the GL is inherited from scraping over the bedforms and is later modified into terraces.

**Extended Data Fig. 5:**
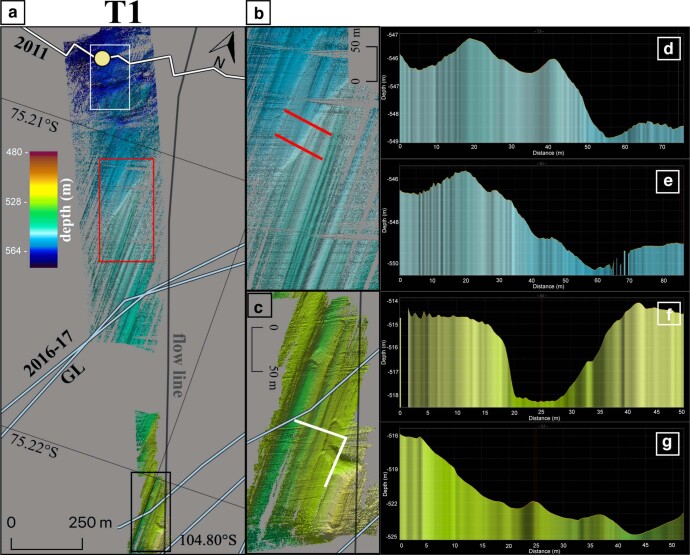
Ridged bedforms with pronounced ridge-crest amplitudes are found along the T1 survey. **a**, Sonar-derived bathymetry from the borehole to the nearest approach made to the GL, coloured by depth (after Fig. 2, bathymetry and profiles produced with Qimera and projected in QGIS), in which boxes denote the sections shown in **b** and **c**. **b**,**c**, Close-up views showing details of two regions of the survey. Red (**b**) and white (**c**) lines denote the position of profiles found in **d** and **e**. **d**,**e**, Linear profiles of the bed topography showing that small-scale ridges formed across larger, longer-wavelength topography. **f**,**g**, Nearest the GL, 1-m, 5-m and 10-m wavelength along-flow ridges are observed.

**Extended Data Fig. 6:**
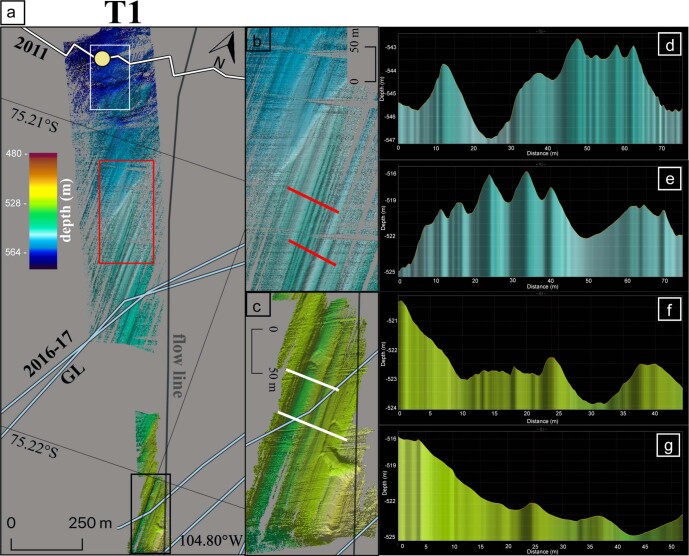
Sea-floor topography suggests past dynamics influencing the retreat of Thwaites Glacier. **a**, Sonar-derived bathymetry from the borehole to the nearest approach made to the GL, coloured by depth, with callouts for panels **b** and **c** (after Fig. 2, bathymetry and profiles produced with Qimera and projected in QGIS). **b**,**c**, Close-up views showing details of two regions of the survey, after Fig. 2. Red and white lines indicate the position of profiles found in **d**–**g**. **d**,**e**, Linear profiles of a crosscutting approximately 3-m-tall sinuous ridge are consistent with a past grounding event allowing enhanced sediment deposition in this area. This is the only such feature in the survey data. **f**,**g**, Linear profiles across a possible former subglacial channel near the GL. In **f**, a 5-m-deep U-shaped trough in the sediment begins parallel to ice flow but then cuts perpendicular to ice flow across lineated bed features and then turns sharply, which could be consistent with a channel incised by subglacial outflow into the sediment. In **g**, the upstream extent of the trough is less conspicuous, suggesting either modification as the ice ungrounded or that the SGW was not routed discretely through this area.

The sea floor was primarily characterized by bedform ridges oriented north–south parallel to glacier flow (Fig. 2). Sea-floor ridge–crest spacing varies by an order of magnitude from 3–25 m and tens of centimetres to 10 m heights; most ridges have 0.5–2 m heights (Extended Data Figs. 4–6). Sporadic boulders and drop stones are visible through the sediment (Fig. 3 and Supplementary Video 1). Near the borehole, troughs crosscutting the ridges suggest reworking of the sediment, which could occur if the glacier was pinned near this location that coincides with the estimated 2011 GL position^12^ (Fig. 2a). Upstream of the borehole, a single semi-linear feature cuts across the along-flow ridges and crests, with a sharp step in depth of 2–3 m height (Fig. 2b); this is downstream of all estimated GL positions for 2016–2017 (ref. ^12^). We interpret this feature as the sediment wedge produced when the ice shelf was grounded at this position, approximately 1,250 m from the end of the T1 survey and 1,500 m from the furthest upstream 2017 GL location. We observe no other clear evidence of GL wedges in this region. Thus, the bathymetry suggests that the GL retreated smoothly up the prograde sea floor, with only one stable location since at least 2011.

Local variations in ice-shelf basal slope (topography) influence melting through modulation of near-ice ocean-density gradients (stratification) and small-scale turbulence that control ocean heat and salt fluxes^32–38^. Nearest the GL, the ice base comprises a system of short-wavelength ridges (Fig. 2e) that have similar shape and 2–5-m spacing of small-amplitude (0.1–0.5 m) ridges in the sea floor (Fig. 2b–d and Extended Data Fig. 4) that overlay broad (about 50 m) topographic undulations. Within a kilometre of the GL, the ice surface is very rough, about 30% consisting of high slopes. Relatively clear ice laden with sediment, called basal ice, is found consistently in this region and in patches downstream, interrupting white, bubble-rich meteoric ice. The fine-grained (sand to mud) debris (Fig. 3a, right and Supplementary Video 1) and interspersed angular clasts ranging in size from a few to tens of centimetres comprises strong laminated layers in the basal ice at centimetre-scale spacing. Visible melting was observed throughout the region, with grains and small drop stones steadily falling out of basal ice, adding to the turbidity of the water column (Supplementary Video 1). Small terraces and scalloped morphology carved into the ice appear within 200 m of the GL, indicating that melting rapidly erodes these sloping ice faces. The steep faces grow in vertical scale with distance from the GL, showing progressive evolution of the shape of the ice from melting the longer it is exposed to the warm ocean.

**Fig. 4:**
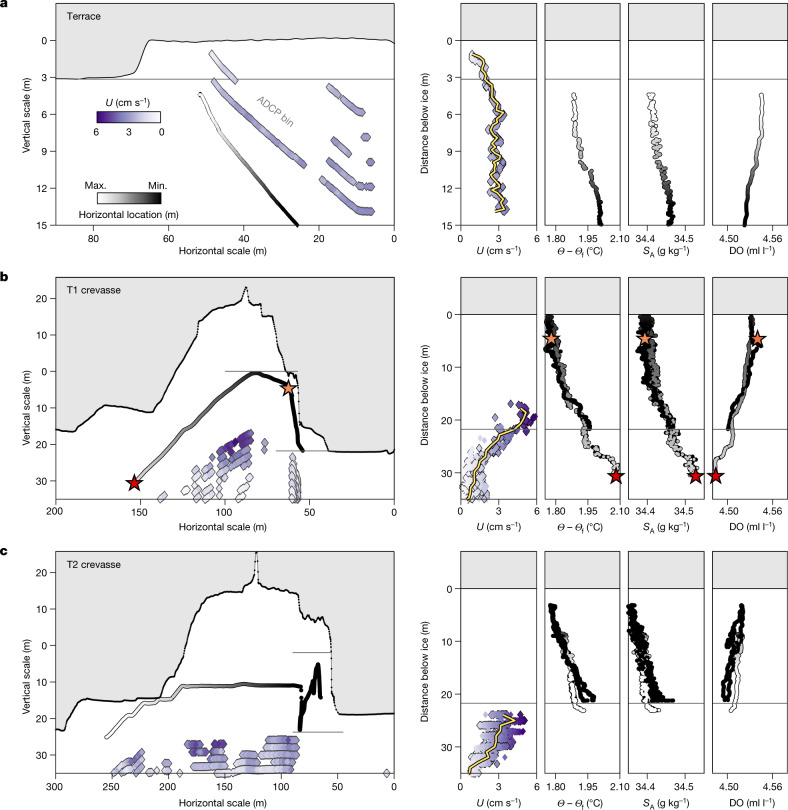
Ocean currents and ice topography contribute to variable melting in terraces and crevasses. Here the Icefin vehicle track is shaded by relative along-track distance from downstream (white) to upstream (black) and current velocities are shaded from slowest (white) to fastest (purple). **a**, Horizontal and vertical trends near a corner of a wide terrace (1,900 m downstream in T1 near the borehole) show freshening and cooling water inside the terrace and slowing currents as the water feels the influence of the ice interface. The grey lines denote the bottom of the terrace. Vertical profiles of ocean-current speed (*U*), thermal driving (*Θ* − *Θ*_f_), absolute salinity (*S*_A_) and dissolved oxygen (DO) binned with distance from the ice base show that, although the water is warm close to the interface, the current velocity slows in the boundary layer, suggesting breaking from friction at the interface^28^. **b**,**c**, As in **a** for the furthest crevasse from the GL, observed along both T1 (**b**) and T2 (**c**). The panels on the right are binned with distance from the top of a step in the crevasse sidewall along T1 marked with the upper grey line. The lower grey line indicates the elevation of the bottom of the crevasse in T1. Stars in **b** relate to the location in the left panel. These panels show warm water with thermal driving of nearly 1.8 °C (*Θ* − *Θ*_f_) reaching the crevasse walls accompanied by very slight freshening and oxygen increase that indicate melting (*S*_A_ and DO) that would then rise into the crevasse.

**Fig. 5:**
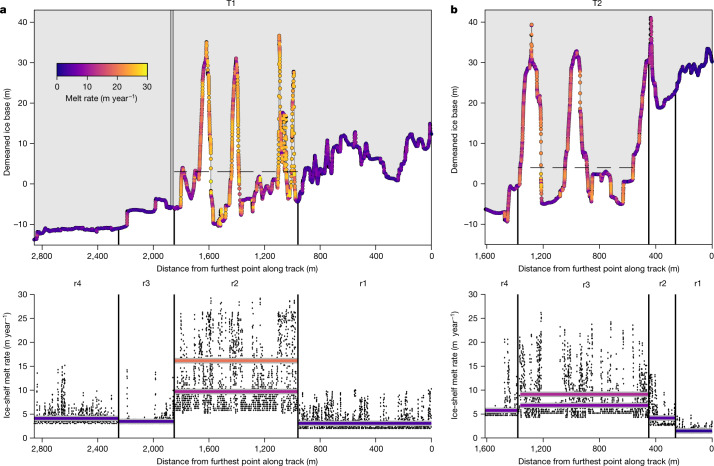
Highly variable melt rates are found beneath the TEIS. **a**,**b**, Estimates of the spatially varying ice-shelf melt rate are shown for each of four subregions along T1 (**a**) and T2 (**b**) (r1–r4 are the same regions as in Extended Data Table 2). The ice surface is coloured by melt rate calculated along each slope (top panels) from the three-equation parameterization (Methods) under regionally averaged ocean conditions, demonstrating the increased melt rate along steep slopes. Horizontal coloured lines (bottom panels) correspond to the mean melt rates in each region. For regions r2 in T1 and r3 in T2, two means are presented, as conditions were observed to change with height in the crevasses, in which the water higher in the crevasses was colder and fresher than the water lower in these features. The lower bar indicates the melt rate determined by variable ocean forcing in the upper crevasse above the dashed lines in the top panels; the upper bar represents the mean melt rate below the dashed line in the crevasses. The means for each of these regions are as follows: T1: r1: 3.07 m year^−1^; r2: 16.16 m year^−1^ (below dashes), 9.72 m year^−1^ (above dashes); r3: 3.48 m year^−1^; r4: 4.11 m year^−1^; T2: r1: 1.47 m year^−1^; r2: 4.18 m year^−1^; r3: 9.12 m year^−1^ (below dashes), 6.82 m year^−1^ (above dashes); r4: 5.76 m year^−1^.

The rough ice base observed at the GL erodes downstream, giving way to steep-sided, flat-roofed terraces (Figs. 1 and 3). The walls of these features form up to 90° angles to their flat roofs and keels, rising tens of centimetres to more than 6 m in height (Fig. 1b,c) and uniformly exhibiting scalloped surface textures (Fig. 3b, right), indicative of turbulent ocean-driven melting^33^. Terraces are also observed in crevasses. Conversely, the downstream ice under the TEIS is extremely flat, with surface slopes less than 5° (Figs. 1, 4 and 5). Ice-shelf basal topography carved by melt has been observed elsewhere, such as keels and channels^29,39–42^, including terraces at nearby Pine Island Glacier^29^ associated with steep slopes along marginal and channel features that were argued to form by means of feedback between slopes and melting. We observed terraces distributed across the TEIS, in many different orientations and across a range of horizontal and vertical scales, both associated with and independent of other basal features. Our observations argue that terraces are widespread basal features of ice shelves that overlay warm ocean cavities; these are not yet represented in most ice-shelf models.

## Ice–ocean interactions

In situ observations of the undisturbed ice–ocean boundary layer beneath ice shelves are inherently difficult to make through boreholes owing to contamination from the heated freshwater used to drill the access hole. Before this work, no in situ measurements existed that could constrain behaviour at the GL. To make these observations, we drove Icefin along the base of the ice to capture the boundary layer along flat interfaces, at an angle towards and then contacting the ice to measure gradients up to the ice, and straight into vertical sidewalls, in some cases measuring within about 5 cm of the interface.

Throughout the region, thermal driving was about 1.75 °C within 1 m of the ice base, providing ample heat to drive melting (Methods). Generally, the near-ice water column under the TEIS closely fits the well-defined mixing lines between GMW and a source water mass, and observations imply fully developed turbulent mixing^35,37^ (Methods and Extended Data Fig. 3), although data nearest the ice reflect increased melt. Our observations show strong vertical stratification approaching flat portions of the ice–ocean interface containing GMW formed from melting along neighbouring slopes rising to the ice base (Figs. 3 and 4). Ocean currents weaken within 5 m of the ice from a background speed near 3 cm s^−1^ (ref. ^28^) to near zero close to the interface (Fig. 4a). By contrast, currents increased in crevasses to a measured maximum of 5.90 cm s^−1^ (Fig. 4b,c).

In the terraces, dissolved oxygen increases with decreasing temperature and salinity, consistent with input from melting ice. Some of the strongest stratification we observed was in a shallow terrace formed along the roof of another large terrace, at which the salinity of the boundary layer was 20 g kg^−1^, or roughly one-third fresher than the surrounding ocean water. Extremely fresh layers (36–42% freshwater) in recesses along terrace roofs are not fully turbulent, as salinity and dissolved oxygen exhibit much larger signatures than temperature, suggesting a regime in which diffusive processes control heat and salt flux^43^. The thicknesses of these fresher layers are on the order of tens of centimetres and probably reflect the transition between the fully turbulent outer and viscous inner portions of the ice–ocean boundary layer^44^.

**Extended Data Fig. 7:**
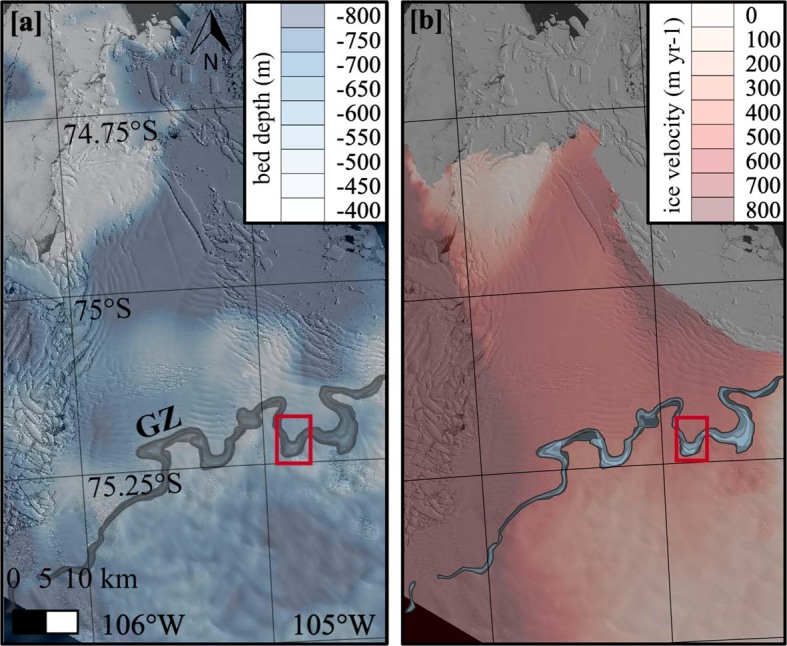
Bed elevation and ice speed for the TEIS show a changing system susceptible to GL retreat. **a**, Colourized bed elevation from BedMachine v3 (ref. ^64^) for the TEIS is overlaid on a Landsat 8 image with the historical GL positions (coloured lines/zones after ref. ^12^ are identical to Fig. 1a and Extended Data Fig. 2a, in which green is 2000, white is 2011 and blue is 2016–2017). QGIS map: Landsat 8, 15 m pixel^−1^, band 8 image LC08_L1GT_003113_20200131_20200211_01_T2_B8, 31 January 2020; the red box denotes the study region. Note that the regions upstream of the present GL are grounded more than 800 m below sea level. **b**, As in **a** except coloured by the average 2019 seaward ice-flow speeds for this region from the 120 m pixel^−1^ ITS_LIVE remote-sensing product^66^. Note that the flow speeds increase as the ice crosses the GL and decrease near the pinning point.

The water closest to the GL is cooler and fresher than the surrounding ocean (excluding freshwater in terrace roofs), with a dissolved oxygen signature distinct from elsewhere in the region. These data have a shallower temperature–salinity (*T*–*S*) slope of 2.05 °C (g kg^−1^)^−1^ than the melt mixing line (roughly 2.5 °C (g kg^−1^)^−1^) and decrease in dissolved oxygen with freshening (Extended Data Fig. 3). This admixture of fresh, oxygen-poor water suggests the presence of discharged SGW from upstream of the GL^45^. Although no SGW source is observed directly, bathymetry near the GL along T1 suggests a recent subglacial channel (Fig. 2c), and SGW outflow measured downstream varies over time^28^. Estimates of SGW concentration calculated from *T*–*S* and DO–*S* properties indicate maximum values of 7 ml l^−1^ and 24 ml l^−1^, respectively. The much higher SGW estimate implied from DO–*S* suggests that the debris-laden basal ice prevalent near the GL is also low in oxygen (such as ref. ^45^) and originated as SGW that was accreted in the overdeepened basin further upstream (Extended Data Fig. 7).

**Fig. 6:**
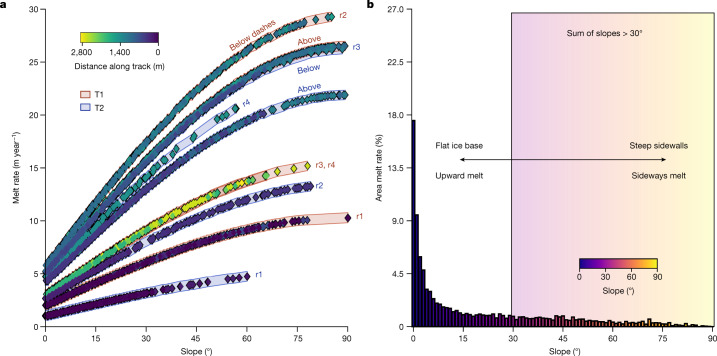
The ice-shelf melt rate is strongly slope-dependent and steep slopes contribute up to 27% of the ice loss under the TEIS along only 9% of the ice base. **a**, Estimated spatially varying ice-shelf melt rates along T1 and T2 show the strong influence of local slope. Here each curve consists of individual melt-rate data points that have been calculated using the regionally averaged ocean conditions (Methods) corresponding to the regions labelled in Fig. 5. Red curves are from T1 and blue curves are from T2. **b**, Sideways melting along slopes greater than 30° contributes an estimated 27% of the melting under the TEIS, whereas these slopes account for only 9% of the ice base. Upward melting along low slopes is still the most notable source of melting, in which slopes less than 30° account for 73% of melting, while representing 91% of the ice.

To test the impact of melting in the region, we calculated melt rates assuming shear-driven turbulent mixing, according to local-ice base slope and using current speeds and hydrographic conditions that were averaged over regions with similar conditions (denoted in Fig. 5). We compared these to results from three autonomous phase-sensitive radio echo sounders (ApRES) and the oceanographic mooring at the borehole^28^ (Fig. 5 and Methods). This approach using regionally averaged ocean conditions (Methods) yields average upward melt rates of 5 m year^−1^, but melt in the region is highly variable (Figs. 5 and 6). The stratification suppresses melting along flat interfaces, whereas estimated melt rates along vertical faces approach 30 m year^−1^.

**Extended Data Fig. 8:**
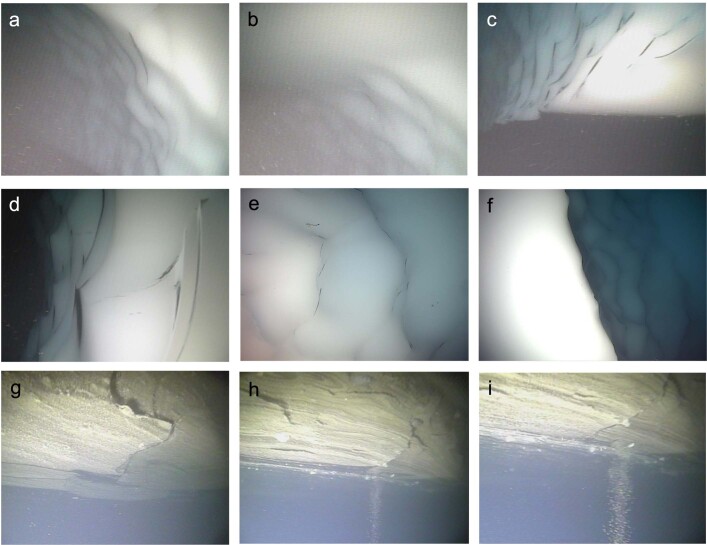
Topography of terraces under the TEIS demonstrates strongly asymmetric melting and melt processes. **a**–**d**, Images of a terrace upstream of the borehole along T1 show a steep, curved sidewall (**a**), flat roof (**b**), sharp transition from the wall to the flat base (**c**) and close-up of scallops in the wall show differences between modes of melting upward along the roof and base with sideways turbulent melting along the sidewall. Upward-looking views of the base (**e**) and sidewall (**f**) of another terrace downstream of the borehole show similar features. Small terraces near the GL along T1 show the initiation of scallops along smaller features in the basal ice (**g**), in which asymmetric ice melting is clear from the shape and streams of particulates (**h**,**i**).

Although stratification suppresses melting upward (moderate vertical melt or thinning), higher lateral turbulent mixing^46^ and destabilizing rising GMW^36,47,48^ allows warm water to reach sloped surfaces and promote melting (high sideways melt; Figs. 5 and 6). The scalloped ice surfaces observed only on steep faces is consistent with high sideways melting (Fig. 3b and Extended Data Fig. 8). Melting is strongest along the near-vertical walls of crevasses, at which water 1.8 °C above freezing was observed reaching within 1 m of the vertical crevasse wall (Fig. 4b). Water cools with height in the middle of the crevasses, freshens and becomes more oxygenated, suggesting a local accumulation of meltwater exceeding 3 ml l^−1^ from the erosion of the crevasse walls. Currents were faster in the crevasses by up to a factor of two compared with the background^28^, with flow speeds reaching about 6 cm s^−1^. These observations imply melt rates along the crevasse sidewalls of up to 43 m year^−1^ in one crevasse at the location of these observations (Methods), whereas melting elsewhere is more suppressed (Fig. 6; ref. ^28^).

## Topographic controls on ice-shelf evolution

These results indicate that ice–ocean interactions under the TEIS are influenced by even small-scale ice topography, which would extend to other warm-based ice shelves in which low to moderate current speeds allow high levels of near-ice ocean stratification to persist. We calculate moderate average upward melting along flat surfaces at 5 m year^−1^, which matches measured melt rates on similar interfaces from three long-term ApRES and mooring data^28^ and are consistent with the historical estimates from ice-penetrating radar^18^. Nearest to the GL along each survey line, melt rates average 2 m year^−1^ but range from 1 to 10 m year^−1^ (Figs. 5 and 6). Our observations show that the feedback between ice slope and melting is relevant to the entire base of ice shelves, including near the GL. The varied topography of the ice base at the GL, carved as it flowed over the bed before reaching the ocean, becomes a broadly distributed network of sloped ice surfaces along which melting is promoted.

Our observations suggest that melting along sloped ice is an important factor in the total ice loss near the GL of Thwaites Glacier. In the region surveyed, 27% of the total melting occurs along slopes that are greater than 30° (Fig. 6). Because crevasses funnel water through them^28^ at rates that can efficiently transfer heat and salt into the steep crevasse walls (Fig. 4), these locally high melt rates should widen both crevasses and basal rifts across the glacier, including the TGT and the TEIS, and could contribute to increased calving of the glacier^8,10^. The rough topography near the GL may enable melting to persist in this region despite low current speeds. Our work implies that basal melting of warm-based ice shelves is heterogeneous and exploits ice topography inherited from interactions with the bed and formed by crevassing. Such effects are challenging to observe, not yet captured in models of GL retreat and probably contribute to the loss of ice elsewhere along the Antarctic coast.

## Methods

### Icefin vehicle

The Icefin vehicle^49^ is a modular, hybrid, remotely operated vehicle with autonomous capabilities that was designed for use through holes bored or melted in ice (Extended Data Fig. 1). For this work, Icefin was fitted with sensors for scientific analysis of the ice–ocean system and navigation (Extended Data Table 1). Most water-column sensors are located in the vehicle nose to provide undisturbed water flow. Forward cameras, lights and sonar provide perception for science and navigation, and bathymetric sonar maps sea-floor geometry. A high-definition camera and Doppler velocity logger (DVL) with acoustic Doppler current profiler (ADCP) point in the same sense, and altimeter in the opposite sense, in the navigation module that can be oriented down (sea-floor facing) or up (ice facing). A rear-facing camera monitors the tether/tail. Guidance, navigation and control of Icefin allow for geolocated scientific data through the fusion of an advanced fibre-optic gyroscope inertial measurement unit, compass, DVL, altimeter and pressure sensor providing low-level motion control and high-level localization. The five thrusters provide five degrees of freedom movement, control pitch, yaw, heave and sway without protruding surfaces, and permit hovering. Icefin is rated to 1.5 km depth, weighs 120 kg in air, is 23 cm wide and 4.5 m long. At the TEIS, Icefin was deployed vertically from an articulating launch and recovery system, lowered through the borehole by means of a 3.5-km-long 4-mm-diameter Kevlar-reinforced (1,800 lb breaking strength; Linden) fibre optic attached to a strength-enforced termination at the vehicle, enabling control, communication and data retrieval.

Icefin was deployed for five 6–8-h missions during 11–14 January 2020 to map environmental gradients in cross-section extending seaward from the GL, overlapping surface, airborne and ApRES surveys. The Icefin data are grouped into two composite profiles, transect 1 (T1) and transect 2 (T2); T1 consists of missions 1 (about 1.2 km south), 2 (about 1.9 km south) and 3 (about 1 km north) and T2 is mission 4 (about 1.6 km southeast). Missions 1-4 were conducted with the navigation module down and mission 5 with the navigation module up. Multipath in the clear basal ice reduced vehicle positioning accuracy in mission 5. T2 intersected the GL at 104.780° W, 75.214° S. For missions 1 and 3, the vehicle conducted survey segments alternating between pitching up towards the ice and down towards the sea floor at about 20–30° to acquire hydrographic profiles while making forward progress, similar to ocean gliders.

### Post-processing of hydrographic data

Hydrographic data come from three sensors on Icefin: conductivity–temperature (*C*–*T*), pressure (*P*) and DO sensors. All sensors were factory calibrated before the fieldwork. *C*–*T* and DO sensors were field calibrated. Pressure measurements (1 Hz) were interpolated to match the 5-Hz *C*–*T* data to derive hydrographic variables. DO measurements (1 Hz) were not interpolated.

#### Data post-processing

1. Remove background atmospheric pressure,
2. Remove outliers ±2 standard deviations from mean for *C*–*T* and DO (excluding borehole data),
3. Apply three-point weighted mean filter to *C*–*T*, *P* and DO,
4. Align *C* and *T* measurements with time lag (0 s lag produced the best results),
5. Remove *C*, *T* and DO data for vehicle speeds <5 cm s^−1^,
6. Derive hydrographic variables (conservative temperature, absolute salinity, density and so on) using TEOS-10 (ref. ^50^),
7. Remove pressure/salinity effects on DO^51^.

### Post-processing ocean-current speeds

Ocean-current speeds are derived from the onboard DVL/ADCP, which calculates the *X*, *Y* and *Z* vehicle velocities (major, minor and vertical axes) and retrieves water-column velocities in 2-m bins at a variable start distance from the vehicle. The minimum altitude from an interface for current profiling to occur is 10 m; gradients in vehicle pitch, roll, heading and speed dictate the distance of the first bin from the vehicle and sampling frequency (maximum 5 Hz). We subsample velocities to 1 Hz. Water-column *X* velocities are differenced from the vehicle velocity, resulting in an uncertainty of ≤1 cm s^−1^. Vehicle *Y* and *Z* velocities are substantially lower than *X*, so *Y* and *Z* velocity uncertainties are likewise lower. Here we only analyse *X* and *Y* velocities.

Data post-processing:

1. Remove data in out-of-range bins (for example, below sea floor, above ice base),
2. Remove spurious data: exactly 0 m s^−1^ or 32,767 m s^−1^,
3. Remove measurements when vehicle pitch or roll >+/−30°,
4. Convert from vehicle reference frame to geographic reference frame,
5. Apply 30 s mean filter,
6. Filter for gradients <1 standard deviation from mean vehicle speed, pitch, roll and individual bin velocity,
7. Collate bins by each up/down vehicle swoop into 1-m vertical bins, removing data >1 standard deviation of the mean for that range.

### Post-processing of ice and sea-floor elevations

Ice-base and sea-floor elevations are derived from DVL and altimeter data and bathymetric sonar data. The DVL takes into account pitch, roll and heading when producing ranges and the altimeter and sonar data are corrected for these attitudes. Bathymetric sonar was processed in Qimera, in which obvious outliers were filtered or cleaned by hand.

Data post-processing (DVL, altimeter):

1. Remove data >2 standard deviations of the mean gradient in interface elevation (ice base or sea floor),
2. Manually remove outliers.

After post-processing, 94% of ice-base measurements had horizontal resolution 26 ± 14 cm, with minimum and maximum spacings of 1.4 mm and 3.38 m, respectively. Ninety-three percent of sea-floor data had horizontal resolution 29 ± 20 cm and minimum and maximum spacings of 2.3 mm and 4.72 m, respectively.

### Water-mass partition

We use a three-point endmember partition^35^ to estimate concentrations of water masses below the shelf, which assumes that hydrographic properties (*Θ*, *S*_A_ and DO) reflect a mixture of three water masses:

1. A source water mass (SRC) responsible for driving melt: *Θ* = −0.21 °C, *S*_A_ = 34.50 g kg^−1^, DO = 4.48 ml l^−1^,
2. GMW from local ice-shelf melt: *Θ* = −92.50 °C, *S*_A_ = 0 g kg^−1^, DO = 25.20 ml l^−1^,
3. SGW discharged from upstream beneath the grounded glacier^52^: *Θ* = −0.34 °C, *S*_A_ = 0 g kg^−1^, DO = 1.61 ml l^−1^.

This partition uses conservative tracers that only vary as a result of a physical mixture of water masses and mix under fully turbulent conditions^35,37^. Subsets of the data not in a fully turbulent mixing regime are excluded from the partition. These data are easily identified by double-diffusive characteristics—large changes in *S*_A_ (and DO) relative to *Θ* that result from faster molecular diffusion of heat than salt^43,44^.

The source water mass (SRC, red star in Extended Data Fig. 3 and Fig. 4b) is the warmest, saltiest and most oxygen-depleted point in the *T*–*S* and DO–*S* space that fits the GMW mixing line on which our data lie: *T*–*S* and DO–*S* slopes of −2.49 °C (g kg^−1^)^−1^ and 0.60 ml l^−1^ (g kg^−1^)^−1^, respectively. SRC generally resides 10 m below the ice base (outside crevasses). Warmer, saltier and more oxygen-depleted data (red data in Extended Data Fig. 3) further down in the water column exhibit a different slope in the *T*–*S* (−2.74 °C (g kg^−1^)^−1^) and DO–*S* (0.35 ml l^−1^ (g kg^−1^)^−1^) space, suggestive of mixing processes not derived from local glacial modification. Thus, SRC hydrographic properties represent the local water mass melting the ice shelf, referred to as a composite tracer^35^. SRC is a derivative mixture of modified CDW and winter water that resides around the depth of the highly variable pycnocline separating these water masses in Pine Island Bay. Relatively weak inflow of about 3 cm s^−1^ into our study region and at a distance of about 75 km from Pine Island Bay^28^ results in a ventilation period of around one month for SRC. During this time *Θ*, *S*_A_ and DO properties could be altered through interaction with the ice base along other sections of the TEIS, consumption by organisms or mixture with other water masses. We forgo choosing hydrographic properties for the absolute source water mass to ensure that the properties used in our three-point water-mass partition are conservative for local ice–ocean interactions.

The *Θ* and *S*_A_ values for GMW consider the latent heat loss associated with phase change from solid to liquid freshwater. An extrapolation of the Gade or GMW mixing line^35,37^ for our data to 0 g kg^−1^ results in an effective temperature of −86.46 °C. GMW is high in DO because of air bubbles in meteoric ice that dissolve into solution when melted. An extrapolation of the DO–*S* mixing line to 0 g kg^−1^ for our data results in a DO concentration of 25.20 ml l^−1^.

The *Θ* and *S*_A_ values for SGW represent freshwater at the pressure-depressed freezing point at GL depth of 480 m in T2 (Fig. 1c). We use in situ DO measurements from Lake Whillans, West Antarctica^45^ for the SGW endmember, as active subglacial lakes exist upstream of the TEIS^53–55^. Thus, we expect that basal ice here should carry similarly low DO.

### Three-equation melt parameterization

We estimate ice-shelf melt rates along T1 and T2 using the three-equation boundary layer (BL) parameterization for heat and salt transfer between the ocean and ice^56^, which assumes that shear-driven turbulence controls ice melt as the dominant mechanism conveying heat and salt to the viscous sublayer (VSL), beyond which molecular diffusion operates^57^. Near-ice ocean-current measurements exhibit shear (Fig. 4a), which agrees with the physics governing Ekman layers^28^ and is consistent with shear-driven turbulence.

This assumption does not hold for regions with low current speeds, at which molecular processes and diffusive convection dictate heat and salt fluxes at distances greater than the typical VSL^44,58^, order several millimetres^56^. We only observed such conditions in two small regions along the roofs of two terraces with extremely fresh layers (*S* ≈ 20 psu) tens of centimetres thick (Fig. 3b,c) that did not hold fully turbulent mixing lines and exhibited larger salinity gradients than temperature. Other near-ice ocean data did not exhibit these thick, fresh layers but instead gradually cooled and freshened towards the ice throughout the surveyed area. This density change stabilizes the BL beneath regions of the ice base, reducing vertical shear-driven turbulent mixing^59^. Therefore, although shear-driven turbulence dominates heat and salt fluxes, turbulent fluxes are modified by stratification^47,60^. We account for stratification in our melt rates.

The three-equation BL parameterization is as follows:

1 $${T}_{{\rm{B}}}=a{S}_{{\rm{B}}}+b+c{p}_{{\rm{B}}}$$

2 $${Q}_{{\rm{O}}}^{{\rm{T}}}-{Q}_{{\rm{I}}}^{{\rm{T}}}={Q}_{{\rm{Latent}}}^{{\rm{T}}}$$

3 $${Q}_{{\rm{O}}}^{{\rm{S}}}-{Q}_{{\rm{I}}}^{{\rm{S}}}={Q}_{{\rm{Fresh}}}^{{\rm{S}}}$$

Equation (1) represents temperature, practical salinity and pressure at the ice–ocean interface, in which *a* = −5.73 × 10^−2^ °C (g kg^−1^)^−1^, *b* = 9.39 × 10^−2^ and *c* = −7.53 × 10^−8^ °C Pa^−1^ are constants and *T*_B_ is always at the freezing point. *T*_B_ and *S*_B_ are not directly observed and are estimated below.

Equation (2) represents the heat flux balance and equation (3) represents the salt flux balance, in which ${Q}_{{\rm{O}}}^{{\rm{S}}}$ is the ocean salt flux towards the ice, ${Q}_{{\rm{I}}}^{{\rm{S}}}$ is the diffusive salt flux through the ice (0 here) and ${Q}_{{\rm{Fresh}}}^{{\rm{S}}}$ is the freshwater flux from melting. ${Q}_{{\rm{O}}}^{{\rm{T}}}$ is the ocean sensible heat flux into the ice base, ${Q}_{{\rm{I}}}^{{\rm{T}}}$ is the conductive heat flux through the ice shelf and ${Q}_{{\rm{Latent}}}^{{\rm{T}}}$ is the latent heat removed during melting:

4 $${Q}_{{\rm{O}}}^{{\rm{T}}}={\rho }_{{\rm{w}}}{c}_{{\rm{p}}}{u}_{* }{\varGamma }_{{\rm{T}}}(T-{T}_{{\rm{B}}})$$

5 $${Q}_{{\rm{Latent}}}^{{\rm{T}}}={\rho }_{{\rm{i}}}{L}_{{\rm{F}}}\dot{m.}$$

In equation (4), *ρ*_w_ and *T* represent the density and temperature, respectively, of seawater outside the BL/VSL. For seawater, *c*_p_ = 3,974 J kg^−1^ °C^−1^, the friction velocity u_*_ is the kinematic stress at the ice–ocean interface and the heat transfer coefficient *Γ*_T_ describes turbulent mixing of heat across the BL. The density of ice is *ρ*_i_ = 918 kg m^−3^, *L*_F_ = 3.34 × 10^5^ J kg^−1^ is latent heat of fusion and $\dot{m}$ is the ice-shelf melt rate (m year^−1^), in which $\dot{m}$ is positive for melting. We use the quadratic stress formula to relate u_*_ to near-ice current speeds^61^:

6 $${u}_{* }^{2}={C}_{{\rm{D}}}{U}^{2}$$

in which *C*_D_ = 2.2 × 10^−3^ is a dimensionless drag coefficient assumed to be constant^27^ and *U* is the current speed. Without a vertical-ice-column temperature profile, we vary ${Q}_{{\rm{I}}}^{{\rm{T}}}$ as 0.12–0.2 of the magnitude of ${Q}_{{\rm{O}}}^{{\rm{T}}}$ (refs. ^42,62^).

7 $${Q}_{{\rm{O}}}^{{\rm{S}}}={\rho }_{{\rm{w}}}{u}_{* }{\varGamma }_{{\rm{S}}}(S-{S}_{{\rm{B}}})$$

8 $${Q}_{{\rm{Fresh}}}^{{\rm{S}}}={\rho }_{{\rm{i}}}\dot{m}({S}_{{\rm{B}}}-\,{S}_{{\rm{I}}})$$

Equations (7) and (8) resemble equations (4) and (5), in which *S* is salinity outside the BL/VSL, *S*_B_ is the ice–ocean interface salinity and *S*_I_ is the salinity of the ice shelf (considered 0 here). The salt transfer coefficient *Γ*_S_ is much smaller than *Γ*_T_, owing to slower molecular diffusion of salt than heat in the VSL^63^. We consider a range of published values for *Γ*_S_ between $\frac{1}{70}$ and $\frac{1}{25}$ of *Γ*_T_ (refs. ^23,57,59^) and choose the ratio that produces optimal agreement between melt rates derived by the heat flux and salt flux equations.

We estimated melt rates in five subregions based on ice-base characteristics and near-ice ocean conditions *T*, *S* and *U* (Fig. 5). We compiled integrated probability density functions of the ocean conditions in each subregion, then consider the 25th, 50th and 75th percentiles of *T* and *S* within 5 m of the ice base and the 50th, 75th and 100th percentiles of *U* within 10 m of the ice base for each subregion. We select higher percentiles (and therefore speeds) for *U* because observed ocean currents increased towards the ice (ref. ^28^; Fig. 4) before slowing from friction (ref. ^28^; Fig. 4) and because less current data were collected near the ice base. Extended Data Table 2 provides ocean properties entered into equations (4), (7) and (8). We then consider an array of ten values for the ice–ocean interface properties (*T*_B_, *S*_B_) by varying *S*_B_ from the minimum observed salinity to the 75th percentile of *S* for each subregion and then converted to *T*_B_ using equation (1) for the ice-base mean pressure.

We estimate *Γ*_T_ by dividing the published range of the thermal Stanton number (${{C}_{{\rm{D}}}^{1/2}\varGamma }_{{\rm{T}}}$ = 2.18 × 10^−4^ – 1.10 × 10^−3^) from observations beneath ice shelves^24,55^ by *C*_D_, yielding *Γ*_T_ = 4.60 × 10^−3^–2.35 × 10^−2^, then considering that *Γ*_S_ ranges between $\frac{1}{70}$ and $\frac{1}{25}$ of *Γ*_T_. We vary *Γ*_T_ and *Γ*_S_ from their minimum to maximum values based on the sine of the ice-base slope, from 0° to 90°, because slope/roughness of the ice interacts with stratification to produce variable melting^36,48,63–65^, and rising buoyant GMW also destabilizes stratification along steep ice slopes. The equations by which we calculated this are as follows:

9 $${\varGamma }_{{\rm{T}}}={\varGamma }_{{{\rm{T}}}_{\min }}+\left({\varGamma }_{{{\rm{T}}}_{\max }}-{\varGamma }_{{{\rm{T}}}_{\min }}\right)\sin \phi $$

10 $${\varGamma }_{{\rm{S}}}={\varGamma }_{{{\rm{S}}}_{\min }}+\left({\varGamma }_{{{\rm{S}}}_{\max }}-{\varGamma }_{{{\rm{S}}}_{\min }}\right)\sin \phi $$

We compared calculated melt rates to ApRES data at three locations^38^: T1 distance 2,250–2,857 m; region 2: T1 distance 1,810–1,904 m and T2 distance 1,480–1,608 m; and region 3: T1 distance 0–960 m. The set of conditions that produced the best fit to the observed melt rates along the flat (slope <5°) ice base were the 75th percentile of *T* and *S*, the 100th percentile of *U* and nearly the freshest *S*_B_ and warmest *T*_B_, with heat conduction ${Q}_{{\rm{I}}}^{{\rm{T}}}=0.12{Q}_{{\rm{I}}}^{{\rm{T}}}$ and salt transfer coefficient *Γ*_S_ = $\frac{1}{45}$*Γ*_T_ = 1.03 × 10^−4^–5.21 × 10^−4^. The best-fit melt-rate estimates are: region 1: 3.41 m year^−1^ versus 3 m year^−1^ observed; region 2: 4.80 m year^−1^ (T1) and 4.65 m year^−1^ (T2) versus 5 m year^−1^ observed; region 3: 2.37 m year^−1^ versus 2 m year^−1^ observed.

We compared stratified, shear-driven turbulent estimates to those for an assumed diffusive-convective melt rate^47^, considering ${Q}_{{\rm{I}}}^{{\rm{T}}}=0.12{Q}_{{\rm{O}}}^{{\rm{T}}}$:

11 $$\dot{m}=\frac{1}{{L}_{{\rm{F}}}{\rho }_{{\rm{i}}}}\left[{\rho }_{{\rm{w}}}{c}_{{\rm{p}}}{k}_{{\rm{T}}}\left(\frac{0.5\left(T-{T}_{{\rm{B}}}\right)}{3\sqrt{4{k}_{{\rm{s}}}t}}\right)-0.12\left({\rho }_{{\rm{w}}}{c}_{{\rm{p}}}{k}_{{\rm{T}}}\left(\frac{0.5\left(T-{T}_{{\rm{B}}}\right)}{3\sqrt{4{k}_{{\rm{s}}}t}}\right)\right)\right]$$

in which *k*_T_ = 1.40 × 10^−7^ m^2^ s^−1^ is the molecular diffusion (conduction) of heat, *k*_s_ = 1.30 × 10^−9^ m^2^ s^−1^ is the molecular diffusion of salt and *t* is the amount of time that diffusive convection has driven melting. For *t* = 1 h, the diffusive-convective melt rate gives 2.26 m year^−1^ for region 1, 2.35 m year^−1^ for region 2 and 2.06 m year^−1^ for region 3. At *t* = 1 day, the melt rate decreases to 0.46 m year^−1^ for region 1, 0.48 m year^−1^ for region 2 and 0.42 m year^−1^ for region 3. At *t* = 1 week, the melt estimate decreases to 0.17 m year^−1^ for region 1, 0.18 m year^−1^ for region 2 and 0.16 m year^−1^ for region 3. Although diffusive convection melt rates initially resemble those observed, they abate to <10% of the observations within a week as the BL grows. The ApRES melt rate time series do not exhibit this decrease, showing that, although stratification inhibits turbulence, current shear still determines the melt rate along flat and low-sloped regions of the survey area.

Tuning the melt rate uniformly over the survey region overestimates melt along portions of the ice base that are in diffusive regimes and possibly in the unmapped tops of crevasses; however, this is a small fraction of the region surveyed. Ocean properties will cool/freshen with height in the crevasses and current speeds may change compared with the lower 10% surveyed (ref. ^28^; Fig. 4). This approach however underestimates melt rates along steep ice slopes below the upper crevasse sections, because low *S*_B_ (22.14 psu) and therefore high *T*_B_ reduces assumed thermal forcing (*T* − *T*_B_): the ApRES at the borehole measured lateral melt rates of 70 m year^−1^ on the terrace wall (mean slope 79°) at 1,800 m along T1, compared with 26.35 m year^−1^ estimated. Changing to a salinity of 34.28 psu (75th percentile for T1 subregion 3) obtains a melt rate of 45.97 m year^−1^, closer to observed.

We also compared shear-driven melt rates to those from a buoyancy-driven turbulent melt-rate parameterization^36^:

12 $$\dot{m}=7.8{(T-{T}_{{\rm{f}}})}^{1.34}{\sin }^{\frac{2}{3}}\phi $$

in which *φ* is the ice slope and *T* − *T*_f_ is thermal driving outside the BL/VSL. For slope *φ* = 79°, *S* = 34.28 psu and *T* = −0.34 °C (75th percentile for T1 subregion 3), the buoyancy-driven melt-rate estimate is 18.43 m year^−1^, considerably lower than that observed and 45.97 m year^−1^ estimated from shear-driven turbulence, and does not account for ${Q}_{{\rm{I}}}^{{\rm{T}}}=0.12{Q}_{{\rm{O}}}^{{\rm{T}}}$. Ocean conditions at the opposing crevasse wall are warmer (−0.33 to −0.28 °C) and saltier (34.26–34.27 psu) than what we use to estimate melt on the terrace wall, suggesting that conditions are warmer near the ice or vertical velocities increase turbulence, increasing melt.

### Statistics

All statistics were performed in MATLAB. The above sections provide the details of these statistical analyses, with the underlying mean and standard deviation functions being native to MATLAB.

## Online content

Any methods, additional references, Nature Portfolio reporting summaries, source data, extended data, supplementary information, acknowledgements, peer review information; details of author contributions and competing interests; and statements of data and code availability are available at 10.1038/s41586-022-05691-0.

### Supplementary information

Supplementary Video 1 Forward-looking video of Icefin’s approach to the GL of the TEIS shows visual evidence of melting and ice–bed interactions. Clear basal ice with embedded pebbles and stones and laminated sediments is observed through the region (video clips from Icefin top forward camera over the last 200 m towards the GL of the TEIS). Variable melting causes particles and pebbles to fall from the ice. The rough, ridged ice base shows topography inherited from scraping along the sea floor as well as the initiation of small, sinuous terraces with scalloped interfaces. At the end of the video, the water column was less than 50 cm thick and the ice is observed resting on the bed in the distance in front of the vehicle.

## Figures and Tables

**Extended Data Table 1:**
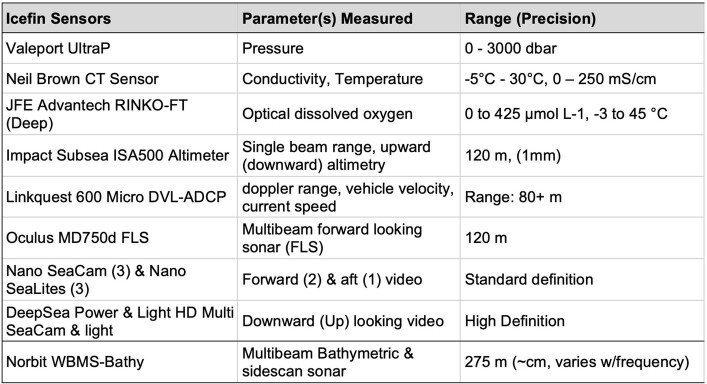
The Icefin vehicle carried 11 science sensors for hydrography and ice and sea-floor measurements during the Thwaites Glacier missions

All temperature, salinity and oxygen data were gathered by sensors in the vehicle forward module (Extended Data Fig. 1). Forward-looking sonar was used to drive the vehicle, providing 100 m forward perception that was used for risk avoidance, mission proximity operations and characterizing ice and sea floor. Forward and up/down cameras and lights characterized the ice base and sea floor when in close proximity. Icefin’s upward (or downward) altimeter and downward (or upward) DVL data (Extended Data Fig. 1) provide along-track ice and sea-floor elevation (Figs. 1–4). In addition to cross comparing with the onboard sensors, Icefin CT profile data from the ice base to the sea floor at the TEIS borehole were compared with CT profile data obtained by a Sea-Bird SBE 49 CTD profiler at the same location taken just before the Icefin dive. These comparisons derive offsets/root-mean-square residuals of C: 0.2862 mS cm^−1^/0.0031 mS cm^−1^ and T: 0.0236 °C/0.0026 °C. The fact that these residuals are lower than the manufacturer-stated accuracies gives confidence in the quality of data post-application.

**Extended Data Table 2:**
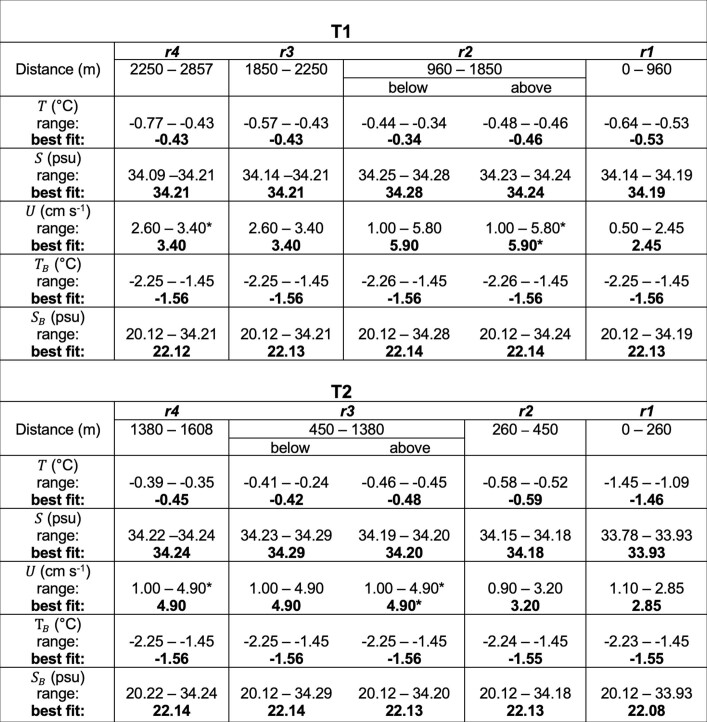
Regionally averaged ocean properties were used to estimate melt rates along T1 and T2

These values were chosen by grouping together under-ice data in which the ocean regime was relatively consistent over hundreds of metres, and then entered into equations (4), (7) and (8) along with each local ice elevation and slope to produce melt-rate estimates. Asterisks indicate areas for which ADCP data were unavailable and thus data collected in another subregion nearby were implemented owing to data paucity. They are as follows: T1 (r4) values taken from T1 (r3), T1 (r2, above dashes) values taken from T1 (r2, below dashes, that is, in the same crevasse), T2 (r4) values taken from T2 (r3, below dashes) and T2 (r3, above dashes) values taken from T2 (r3, below dashes, that is, in the same crevasse).

## Data Availability

The datasets generated and/or analysed during the present study have been submitted to https://www.usap-dc.org/ and are available at 10.15784/601618. The processing and figure-plotting scripts are available at 10.5281/zenodo.7278005.
