# Publisher Correction: Heterogeneous melting near the Thwaites Glacier grounding line

**DOI:** 10.1038/s41586-023-05861-8

**Published:** 2023-02-24

**Authors:** B. E. Schmidt, P. Washam, P. E. D. Davis, K. W. Nicholls, D. M. Holland, J. D. Lawrence, K. L. Riverman, J. A. Smith, A. Spears, D. J. G. Dichek, A. D. Mullen, E. Clyne, B. Yeager, P. Anker, M. R. Meister, B. C. Hurwitz, E. S. Quartini, F. E. Bryson, A. Basinski-Ferris, C. Thomas, J. Wake, D. G. Vaughan, S. Anandakrishnan, E. Rignot, J. Paden, K. Makinson

**Affiliations:** 1 Department of Astronomy, Cornell University, Ithaca, NY USA; 2 Department of Earth and Atmospheric Sciences, Cornell University, Ithaca, NY USA; 3 British Antarctic Survey, Cambridge, UK; 4 Courant Institute of Mathematical Sciences, New York University, New York, NY USA; 5 Center for Global Sea Level Change, New York University Abu Dhabi, Abu Dhabi, United Arab Emirates; 6 School of Earth and Atmospheric Sciences, Georgia Institute of Technology, Atlanta, GA USA; 7 Department of Environmental Studies, University of Portland, Portland, OR USA; 8 Department of Geosciences, Pennsylvania State University, State College, PA USA; 9 Environmental Studies, Lewis & Clark College, Portland, OR USA; 10 Department of Earth System Science, University of California, Irvine, Irvine, CA USA; 11 Center for Remote Sensing and Integrated Systems, University of Kansas, Lawrence, KS USA

**Keywords:** Cryospheric science, Physical oceanography, Physical oceanography

Correction to: *Nature* 10.1038/s41586-022-05691-0 Published online 15 February 2023

In the version of this article originally published, units on the colour bar in Fig. 4a, now reading “6, 3, 0,” were initially jumbled, reading “0, 36”. In addition, the scale was inverted. The units and scale have been corrected in the HTML and PDF versions of the article.

